# Combined lifestyle, childhood trauma and depressive symptoms in adults with subthreshold depression: a prospective cohort study

**DOI:** 10.1017/S2045796025100127

**Published:** 2025-07-15

**Authors:** Yanzhi Li, Yan Chen, Hao Zhao, Wenjing Zhou, Wenjian Lai, Jiejing Hao, Subinuer Yiming, Ruiying Chen, Huimin Zhang, Yuhua Liao, Wanxin Wang, Xue Han, Ciyong Lu

**Affiliations:** 1 Department of Medical Statistics and Epidemiology, School of Public Health, Sun Yat-sen University, Guangzhou, China; 2 Guangdong Provincial Key Laboratory of Food, Nutrition and Health, Sun Yat-sen University, Guangzhou, China; 3 Department of Psychiatry, Shenzhen Nanshan Center for Chronic Disease Control, Shenzhen, China

**Keywords:** childhood trauma, depressive symptoms, healthy lifestyle, modifying role, subthreshold depression

## Abstract

**Aims:**

Existing evidence on the association between combined lifestyle and depressive symptoms is limited to the general population and is lacking in individuals with subthreshold depression, a high-risk group for depressive disorders. Furthermore, it remains unclear whether an overall healthy lifestyle can mitigate the association between childhood trauma (CT) and depressive symptoms, even in the general population. We aimed to explore the associations of combined lifestyle, and its interaction with CT, with depressive symptoms and their subtypes (i.e. cognitive-affective and somatic symptoms) among adults with subthreshold depression.

**Methods:**

This dynamic cohort was initiated in Shenzhen, China in 2019, including adults aged 18–65 years with the Patient Health Questionnaire-9 (PHQ-9) score of ≥ 5 but not diagnosed with depressive disorders at baseline. CT (present or absent) was assessed with the Childhood Trauma Questionnaire-Short Form. Combined lifestyle, including no current drinking, no current smoking, regular physical exercise, optimal sleep duration and no obesity, was categorized into 0–2, 3 and 4–5 healthy lifestyles. Depressive symptoms were assessed using the PHQ-9 during follow-up. This cohort was followed every 6 months, and as of March 2023, had been followed for 3.5 years.

**Findings:**

This study included 2298 participants (mean [SD] age, 40.3 [11.1] years; 37.7% male). After fully adjusting for confounders, compared with 0–2 healthy lifestyles, 3 (*β* coefficient, −0.619 [95% CI, −0.943, −0.294]) and 4–5 (*β* coefficient, −0.986 [95% CI, −1.302, −0.671]) healthy lifestyles were associated with milder depressive symptoms during follow-up. There exists a significant synergistic interaction between a healthy lifestyle and the absence of CT. The CT-stratified analysis showed that compared with 0–2 healthy lifestyles, 3 healthy lifestyles were associated with milder depressive symptoms in participants with CT, but not in those without CT, and 4–5 healthy lifestyles were associated with milder depressive symptoms in both participants with and without CT, with a stronger association in those with CT. The lifestyle-stratified analysis showed that CT was associated with more severe depressive symptoms in participants with 0–2 healthy lifestyles, but not in those with 3 or 4–5 healthy lifestyles. Cognitive-affective and somatic symptoms showed similar results.

**Conclusions:**

In this 3.5-year longitudinal study of adults with subthreshold depression, an overall healthy lifestyle was associated with subsequent milder depressive symptoms and their subtypes, with a stronger association in adults with CT than those without CT. Moreover, an overall healthy lifestyle mitigated the association of CT with depressive symptoms and their subtypes.

## Introduction

According to data from the 2021 Global Burden of Disease Study, depressive disorders affect over 332 million individuals and are the second largest contributor of years lived with disability worldwide (GBD 2021 Diseases and Injuries Collaborators, 2024). From the spectrum perspective, subthreshold depression is a state between health and depressive disorders (Rodríguez *et al.*, 2012). Individuals with subthreshold depression refer to those who have depressive symptoms but do not meet the diagnostic criteria (Rodríguez *et al.*, 2012). A meta-analysis has shown that the prevalence of subthreshold depression is 11.0% inthe general population, and individuals with subthreshold depression have three times the risk of developing depressive disorders as those without (Zhang *et al.*, 2023a). Obviously, individuals with subthreshold depression are a high-risk group for depressive disorders, and identifying modifiable factors alleviating their depressive symptoms is crucial to preventing depressive disorders.

Lifestyles, such as physical activity, smoking, drinking, sleep and body mass index (BMI), are of great concern in disease prevention due to their modifiable nature (Wang *et al.*, 2021). Lifestyles tend to coexist and are interrelated in the real world (Zhang *et al.*, 2021), so exploring the association between combined lifestyle and depressive symptoms is advocated (Cao *et al.*, 2021; Collins *et al.*, 2023; Dabravolskaj *et al.*, 2023; Wang *et al.*, 2021; Werneck *et al.*, 2022). A meta-analysis of observational studies has shown that adherence to an overall healthy lifestyle is associated with a lower risk of depressive symptoms (Wang *et al.*, 2021). Since existing studies have been conducted in the general population (Cao *et al.*, 2021; Collins *et al.*, 2023; Dabravolskaj *et al.*, 2023; Wang *et al.*, 2021; Werneck *et al.*, 2022), it remains unclear whether these findings can be generalized to individuals with subthreshold depression. Moreover, depressive symptoms are highly heterogeneous and are usually divided into somatic symptoms and cognitive-affective symptoms (Iob *et al.*, 2020a). Currently, only a few studies have evaluated the associations between a single lifestyle (i.e. physical activity and BMI) and subtypes of depressive symptoms in the general population, but have ignored combined lifestyle (Chu *et al.*, 2023; Wu *et al.*, 2024). Hence, it is necessary to conduct studies to explore the associations of combined lifestyle with depressive symptoms and their subtypes among individuals with subthreshold depression.

The possible biological mechanisms by which a healthy lifestyle prevents or alleviates depressive symptoms involve maintaining homeostasis of the hypothalamic-pituitary-adrenal (HPA) axis and immune inflammation (Lopresti *et al.*, 2013). Contrary to a healthy lifestyle, childhood trauma (CT) is a recognized risk factor for depressive symptoms (Humphreys *et al.*, 2020), and the biological mechanisms might involve dysregulation of the HPA axis and immune inflammation (Iob *et al.*, 2021, 2020b). These suggest that a healthy lifestyle might mitigate the CT-induced exacerbation of depressive symptoms. In addition, unlike lifestyle, CT cannot be changed once it occurs, and its adverse effects might persist over a lifetime. Therefore, if an overall healthy lifestyle can mitigate or offset the CT-induced exacerbation of depressive symptoms, it is of great significance for preventing depressive disorders among individuals with CT, particularly from a public health standpoint. However, previous studies have only explored the modifying role of a single lifestyle (e.g. physical activity, smoking, alcohol consumption, sleep and BMI) in the association between CT and depressive symptoms among the general population, and the results are mixed (Boisgontier *et al.*, 2020; Jiang *et al.*, 2022; Masuya *et al.*, 2024; Ramirez and Milan, 2016; Rice *et al.*, 2021; Rowland *et al.*, 2023; Royer and Wharton, 2022; Zhang *et al.*, 2023b). To date, it remains unclear whether or to what extent adopting an overall healthy lifestyle can alleviate the CT-induced exacerbation of depressive symptoms, whether in individuals with subthreshold depression or in the general population.

Therefore, this longitudinal study aimed to explore the associations of combined lifestyle, and its interaction with CT, with depressive symptoms and their subtypes (i.e. cognitive-affective and somatic symptoms) among adults with subthreshold depression.

## Methods

### Study design and participants

Data were from the Subthreshold Depression Cohort (SDC), a sub-cohort of the Depression Cohort in China, which was previously described in detail (Zhang *et al.*, 2022). Briefly, the SDC is an ongoing, dynamic and prospective cohort that was launched in 2019. Participants were recruited from 34 primary health care centres in Nanshan District, Shenzhen, China, who were between 18 and 65 years of age, had no past or current psychiatric disorders (e.g. depressive disorders, schizophrenia, social phobia, obsessive-compulsive disorders, generalized anxiety disorders and substance abuse disorders), and were not pregnant or breastfeeding. Participants filled in the Patient Health Questionnaire-9 (PHQ-9) (Spitzer *et al.*, 1999), and those with a PHQ-9 score ≥ 5 would be diagnosed with depressive disorders by a specialized psychiatrist using the Mini International Neuropsychiatric Interview (Diagnostic and Statistical Manual of Mental Disorders, Fourth Edition criteria) (Liao *et al.*, 2023). Participants with a PHQ-9 score of ≥ 5 but not diagnosed with depressive disorders were determined to have subthreshold depression and were included in the SDC (Liao *et al.*, 2023). Depressive symptoms were assessed using the PHQ-9 every 6 months during follow-up. This study was approved by the Institutional Review Board of School of Public Health, Sun Yat-sen University (L2017044). All participants filled in informed consent. All procedures complied with the ethical standards of the relevant national and institutional committees on human experimentation and with the Helsinki Declaration of 1975, as revised in 2013.

As of March 2023, the SDC had been followed for 3.5 years and a total of 2306 participants had participated in the follow-up of this longitudinal study. After excluding participants with missing data on CT (*n* = 0) or lifestyle (*n* = 6), and those without data on depressive symptoms during follow-up (*n* = 2), we included 2298 participants in the analysis (**Figure S1** in the Supplementary).

### Assessment of combined lifestyle

At baseline, lifestyle factors were investigated through the self-reported questionnaire. Referring to previous studies, we defined the following five healthy lifestyles: no current smoking (Jia *et al.*, 2023), no current drinking (Tang *et al.*, 2024), regular physical exercise (Liao *et al.*, 2022), optimal sleep duration (7 to < 9 h) (Lloyd-Jones *et al.*, 2022) and no obesity (BMI < 28 kg/m^2^) (Qie *et al.*, 2024). Regular physical exercise was defined as exercising once a week for at least 30 min each time (Liao *et al.*, 2022). BMI was calculated by dividing weight in kilograms by the square of height in meters. For each lifestyle, we assigned 1 point for a healthy level and 0 point for an unhealthy level. Healthy lifestyle scores were the sum of the points and ranged from 0 to 5, with a higher score indicating a healthier lifestyle. Since a few participants adopted 0, 1 or 5 healthy lifestyles and the lower (33.3%) and upper (66.7%) tertiles of healthy lifestyle scores are 3 and 4, respectively, combined lifestyle was categorized into unfavourable (0–2), intermediate (3), and favourable (4–5) lifestyles.

### Assessment of CT

At baseline, the Childhood Trauma Questionnaire-Short Form (CTQ-SF) was used to investigate CT occurring before the age of 16 (Bernstein *et al.*, 2003). The CTQ-SF has high reliability and validity among the Chinese population (Zhao *et al.*, 2005). The CTQ-SF includes five dimensions (i.e. physical abuse, emotional abuse, sexual abuse, physical neglect and emotional neglect). Each dimension includes five items and each item was rated on a 5-point Likert scale (‘never’ = 1; ‘rarely’ = 2; ‘sometimes’ = 3; ‘often’ = 4 and ‘very often’ = 5). Each dimension score ranges from 5 to 25. Based on the cut-off points suggested by Bernstein *et al.* (2003), we used the following cut-off points for the presence of each CT: physical abuse scores ≥ 10, emotional abuse scores ≥ 13, sexual abuse scores ≥ 8, physical neglect scores ≥ 10 and emotional neglect scores ≥ 15 (Huang *et al.*, 2012; Xie *et al.*, 2023). Participants experiencing one or more subtypes of trauma were considered to have CT (Xie *et al.*, 2023). The CTQ-SF has a high reliability in this study (McDonald’s omega = 0.90).

### Assessment of depressive symptoms

At baseline and follow-up, depressive symptoms were assessed using the PHQ-9 (Spitzer *et al.*, 1999), with high reliability and validity among the Chinese population (Sun *et al.*, 2017). The PHQ-9 includes nine items and each item is scored from 0 to 3 (‘not at all’ = 0; ‘several days’ = 1; ‘more than half the days’ = 2; and ‘nearly every day’ = 3). Cognitive-affective symptoms were evaluated with items 1, 2, 6, 7 and 9, and somatic symptoms were evaluated with items 3, 4, 5 and 8 (Liao *et al.*, 2022; Vrany *et al.*, 2016). Depressive, cognitive-affective and somatic symptom scores range from 0 to 27, from 0 to 15 and from 0 to 12, respectively. A higher score suggests more severe symptoms. McDonald’s omegas for depressive, cognitive-affective and somatic symptoms were 0.88, 0.83 and 0.76, respectively.

### Assessment of covariates

At baseline, covariates were evaluated using the self-report questionnaire. Sociodemographic factors included age, sex (male or female), educational level (junior high school or below; senior high school; or college or above) (Wang *et al.*, 2022), employment status (employed, unemployed. retired or others), marital status (married, unmarried or divorced/widowed) (Liao *et al.*, 2021), and household income (<10 000 yuan/month; 10 000–19 999 yuan/month; or ≥ 20 000 yuan/month) (Shi *et al.*, 2023). Chronic diseases included hypertension, diabetes, heart disease, stroke, thyroid disease and tumors. Since a few participants had more than two diseases, the number of chronic diseases was categorized into 0, 1 and ≥ 2.

### Statistical analyses

Baseline characteristics of patients were summarized across three lifestyle groups. Categorical variables were shown as frequency (percentage) and were compared using the Pearson Chi-squared tests or Fisher’s exact tests, as appropriate. Continuous variables were shown as mean (standard deviation [SD]) and were compared using the one-way analysis of variance or Kruskal-Wallis tests, as appropriate.

The missing proportions of all covariates were less than 0.3% (**Table S1** in the Supplementary). To maximize the statistical power, we performed multiple imputations with chained equations with 10 data sets to impute covariates with missing values. Linear mixed models with random intercepts were used to estimate *β* coefficients and 95% confidence intervals (CIs) to explore the associations of combined lifestyle and CT with depressive symptoms during follow-up. Model 1 was adjusted for follow-up time (follow-up years from baseline) and baseline depressive symptoms (for depressive symptoms), cognitive-affective symptoms (for cognitive-affective symptoms) and somatic symptoms (for somatic symptoms). Model 2 was further adjusted for baseline factors including age, sex, educational level, employment status, marital status, household income and the number of chronic diseases. Model 3 was additionally adjusted for CT (for combined lifestyle) and combined lifestyle (for CT) at baseline. We repeated the above analysis process in the form of a continuous variable for combined lifestyle (each additional healthy lifestyle). Moreover, the dose–response associations of combined lifestyle with depressive symptoms were explored using the restricted cubic spline linked to linear mixed models, with three knots at the 10th, 50th and 90th percentiles of combined lifestyle.

The CTQ-SF measures the CT occurring before the age of 16, which is a relatively long time for elderly adults. Thus, the association between CT and depressive symptoms in young adults might be different from that in elderly adults, that is, age might modify the association between CT and depressive symptoms. We evaluated the interaction between age and CT by establishing a model that included CT, age, CT × age and covariates in model 3. The age-stratified analyses were further conducted if the interaction term (i.e. CT × age) was statistically significant.

To assess the interaction between combined lifestyle and CT, we established a model including combined lifestyle, CT, combined lifestyle × CT and covariates in model 3. Stratified analyses were further conducted if the interaction term (i.e. combined lifestyle × CT) was statistically significant.

To explore the joint associations, we classified participants into six groups according to combined lifestyle (0–2, 3 or 4–5 healthy lifestyles) and CT (yes or no) and estimated *β* coefficients and 95% CIs in different groups compared with those with 4–5 healthy lifestyles and without CT.

To verify the robustness of the results, we conducted three sensitivity analyses. First, we evaluated the association of weighted healthy lifestyle scores with depressive symptoms. Although the simple additive method of combined lifestyle had been used widely (Jin *et al.*, 2020; Zhang *et al.*, 2021), the underlying assumption is that the associations between different lifestyle factors and the outcome were identical, which might not be true. Therefore, we constructed weighted healthy lifestyle scores, where each lifestyle factor was weighted by its association with the outcome (i.e. *β* coefficients in **Table S2** in the Supplementary). Participants were divided into three groups (i.e. unfavorable, intermediate and favorable) based on tertiles of weighted scores (Jia *et al.*, 2023; Zhang *et al.*, 2021). Second, we explored the association of combined lifestyle with depressive symptoms by sequentially excluding each lifestyle to identify lifestyles that might drive the association with depressive symptoms. The excluded lifestyle was used as a confounder. Finally, we performed all analyses in the main analysis after excluding participants with missing values for covariates to test the effect of missing values on the results.

All statistical analyses were conducted using Stata version 17.0 (StataCorp LLC). Statistical significance was defined as a two-tailed *P*-value < 0.05.

## Results

### Characteristics of participants

At the end of 3.5 years follow-up, the proportions of being diagnosed depressive disorders, keeping threshold depression and having no depressive symptoms (i.e. PHQ-9 score < 5) were 9.7%, 37.8% and 52.5%, respectively. Among the 2298 participants included in the analysis, the average age was 40.3 (SD, 11.1) years and 37.7% were male (Table 1). Compared with participants with 0–2 healthy lifestyles, those with 4–5 healthy lifestyles were more likely to be male and retired and were less likely to experience CT and have two or more chronic diseases. In addition, they have higher educational level, higher household income, and lower depressive, cognitive-affective, and somatic symptom scores. There was no statistically significant difference in baseline characteristics between total participants (*n* = 2306) and those included in analyses (*n* = 2298). (**Table S3** in the Supplementary).

**Table 1.** Baseline characteristics of participants with subthreshold depression by combined lifestyle at baseline

Characteristic^a^	Total (*n* = 2298)	0 − 2 Healthy lifestyles (*n* = 408)	3 Healthy lifestyles (*n* = 746)	4 − 5 Healthy lifestyles (*n* = 1143)	*P*-value^b^
Age, mean (SD), years	40.3 (11.1)	39.2 (11.1)	40.8 (11.4)	40.3 (11.0)	0.056
Male	866 (37.7)	275 (67.2)	277 (37.1)	314 (27.5)	<0.001
Educational level					0.001
Junior high school or below	286 (12.5)	51 (12.5)	107 (14.4)	128 (11.2)	
Senior high school	536 (23.4)	123 (30.1)	172 (23.1)	241 (21.1)	
College or above	1472 (64.2)	234 (57.4)	466 (62.5)	772 (67.7)	
Employment status					0.011
Employed	1816 (79.1)	346 (84.6)	577 (77.3)	893 (78.3)	
Unemployed	77 (3.4)	10 (2.4)	30 (4.0)	37 (3.2)	
Retired	156 (6.8)	11 (2.7)	57 (7.6)	88 (7.7)	
Others	247 (10.8)	42 (10.3)	82 (11.0)	123 (10.8)	
Marital status					0.223
Married	623 (27.1)	127 (31.1)	188 (25.2)	308 (26.9)	
Unmarried	1586 (69.0)	269 (65.8)	524 (70.2)	793 (69.4)	
Divorced/widowed	89 (3.9)	13 (3.2)	34 (4.6)	42 (3.7)	
Household income					<0.001
<10 000 yuan/month	1055 (46.0)	217 (53.2)	358 (48.0)	480 (42.1)	
10 000−19 999 yuan/month	685 (29.9)	118 (28.9)	202 (27.1)	365 (32.0)	
≥20 000 yuan/month	553 (24.1)	73 (17.9)	185 (24.8)	295 (25.9)	
Lifestyles					
Regular physical exercise	975 (42.4)	69 (16.9)	191 (25.6)	715 (62.5)	<0.001
Current not smoking	1940 (84.4)	163 (39.9)	661 (88.6)	1116 (97.6)	<0.001
Current not drinking	1563 (68.0)	66 (16.1)	466 (62.5)	1031 (90.2)	<0.001
Optimal sleep duration (7 to <9 h)	1188 (51.7)	92 (22.5)	222 (29.8)	874 (76.5)	<0.001
Body mass index <28 kg/m^2^	2156 (93.8)	327 (80.0)	698 (93.6)	1131 (99.0)	<0.001
Number of chronic diseases					0.041
0	1923 (83.7)	343 (83.9)	612 (82.0)	968 (84.7)	
1	332 (14.4)	52 (12.7)	120 (16.1)	160 (14.0)	
≥2	43 (1.9)	14 (3.4)	14 (1.9)	15 (1.3)	
With childhood trauma	842 (36.6)	178 (43.5)	266 (35.7)	398 (34.8)	0.006
Depressive symptom score, mean (SD)	8.7 (4.3)	9.9 (4.8)	9.5 (4.7)	7.8 (3.6)	<0.001
Cognitive-affective symptom score, mean (SD)	4.2 (2.7)	4.8 (3.0)	4.6 (2.9)	3.7 (2.3)	<0.001
Somatic symptom score, mean (SD)	4.5 (2.2)	5.2 (2.4)	4.9 (2.3)	4.0 (1.9)	<0.001

a Unless otherwise indicated, data are expressed as No. (%) of participants. Percentages have been rounded, so the total may not be 100%.

b One-way analyses of variance were used to compare the means of continuous variables. Pearson Chi-squared tests were performed to compare the distribution of categorical variables.

Abbreviation: SD, standard deviation.

### Individual association of combined lifestyle and CT with depressive symptoms

**Figure 1.**
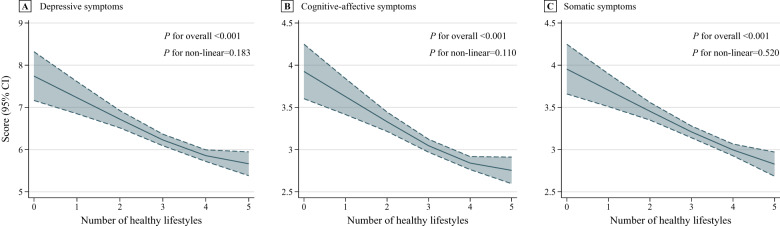
Dose–response associations between combined lifestyle and depressive symptoms during follow-up.

After adjusting for all covariates (Table 2, model 3), compared with 0–2 healthy lifestyles, 3 (*β* coefficient, −0.619 [95% CI, −0.943, −0.294]) and 4–5 (*β* coefficient, −0.986 [95% CI, −1.302, −0.671]) healthy lifestyles were associated with milder depressive symptoms during follow-up. Each additional healthy lifestyle was related to milder depressive symptoms during follow-up (*β* coefficient, −0.381 [95% CI, −0.493, −0.269]). The restricted cubic spline showed a negative linear dose–response association between combined lifestyle and depressive symptoms (Fig. 1a, *P* for overall < 0.001 and *P* for non-linear = 0.183). Similar results were found for cognitive-affective and somatic symptoms (Fig. 1b and 1c). Moreover, CT was correlated with more severe depressive (Table 2, model 3, *β* coefficient, 0.438 [95% CI, 0.222 and 0.654]), cognitive-affective (*β* coefficient, 0.311 [95% CI, 0.183 and 0.440]), somatic (*β* coefficient, 0.124 [95% CI, 0.012 and 0.235]) symptoms during follow-up. We did not observe a modifying role of age in the association between CT and depressive (**Table S4** in the Supplementary, *P*-value for interaction term = 0.162), cognitive-affective (*P*-value for interaction term = 0.055), and somatic (*P*-value for interaction term = 0.451) symptoms.

The solid line and dashed line represent the estimated values and their 95% CI. The adjusted covariates included follow-up time (follow-up years from baseline) and baseline factors, including age, sex, educational level, employment status, marital status, household income, the number of chronic diseases, childhood trauma, depressive symptoms (for depressive symptoms), cognitive-affective symptoms (for cognitive-affective symptoms) and somatic symptoms (for somatic symptoms). The specific locations of the three knots were 2, 4 and 5 healthy lifestyles, respectively.Abbreviations: CI, confidence interval.

**Table 2.** Individual association of childhood trauma and combined lifestyle with depressive symptoms during follow-up

	N	Model 1		Model 2		Model 3	
		*β* coefficient (95% CI)	*P*-value	*β* coefficient (95% CI)	*P*-value	*β* coefficient (95% CI)	*P*-value
**Depressive symptoms**							
Combined lifestyle							
0−2 Healthy lifestyles	409	0 [reference]		0 [reference]		0 [reference]	
3 Healthy lifestyles	746	−0.625 (−0.949, −0.301)	<0.001	−0.658 (−0.984, −0.333)	<0.001	−0.619 (−0.943, −0.294)	<0.001
4−5 Healthy lifestyles	1143	−0.934 (−1.241, −0.627)	<0.001	−1.015 (−1.333, −0.698)	<0.001	−0.986 (−1.302, −0.671)	<0.001
Each additional healthy lifestyle	2298	−0.356 (−0.465, −0.246)	<0.001	−0.390 (−0.502, −0.277)	<0.001	−0.381 (−0.493, −0.269)	<0.001
Childhood trauma							
No	1456	0 [reference]		0 [reference]		0 [reference]	
Yes	842	0.466 (0.243, 0.688)	<0.001	0.462 (0.245, 0.680)	<0.001	0.438 (0.222, 0.654)	<0.001
**Cognitive-affective symptoms**							
Combined lifestyle							
0−2 Healthy lifestyles	409	0 [reference]		0 [reference]		0 [reference]	
3 Healthy lifestyles	746	−0.355 (−0.537, −0.173)	<0.001	−0.366 (−0.549, −0.184)	<0.001	−0.342 (−0.524, −0.160)	<0.001
4−5 Healthy lifestyles	1143	−0.531 (−0.702, −0.360)	<0.001	−0.571 (−0.748, −0.394)	<0.001	−0.554 (−0.730, −0.378)	<0.001
Each additional healthy lifestyle	2298	−0.199 (−0.259, −0.138)	<0.001	−0.217 (−0.279, −0.154)	<0.001	−0.211 (−0.274, −0.149)	<0.001
Childhood trauma							
No	1456	0 [reference]		0 [reference]		0 [reference]	
Yes	842	0.278 (0.153, 0.404)	<0.001	0.279 (0.157, 0.401)	<0.001	0.265 (0.143, 0.387)	<0.001
**Somatic symptoms**							
Combined lifestyle							
0−2 Healthy lifestyles	409	0 [reference]		0 [reference]		0 [reference]	
3 Healthy lifestyles	746	−0.292 (−0.456, −0.127)	<0.001	−0.323 (−0.489, −0.158)	<0.001	−0.305 (−0.470, −0.140)	<0.001
4−5 Healthy lifestyles	1143	−0.509 (−0.665, −0.353)	<0.001	−0.558 (−0.719, −0.396)	<0.001	−0.543 (−0.704, −0.382)	<0.001
Each additional healthy lifestyle	2298	−0.203 (−0.258, −0.147)	<0.001	−0.220 (−0.278, −0.163)	<0.001	−0.216 (−0.273, −0.159)	<0.001
Childhood trauma							
No	1456	0 [reference]		0 [reference]		0 [reference]	
Yes	842	0.214 (0.101, 0.327)	<0.001	0.208 (0.098, 0.319)	<0.001	0.194 (0.084, 0.304)	0.001

Model 1: adjusted for follow-up time (follow-up years from baseline) and baseline depressive symptoms (for depressive symptoms), cognitive-affective symptoms (for cognitive-affective symptoms), and somatic symptoms (for somatic symptoms). Model 2: model 1 plus baseline factors including age, sex, educational level, employment status, marital status, household income, and number of chronic diseases. Model 3: model 2 plus childhood trauma (for combined lifestyle) and combined lifestyle (for childhood trauma) at baseline. Abbreviations: CI, confidence interval.

### Interaction of combined lifestyle and CT on depressive symptoms

**Figure 2.**
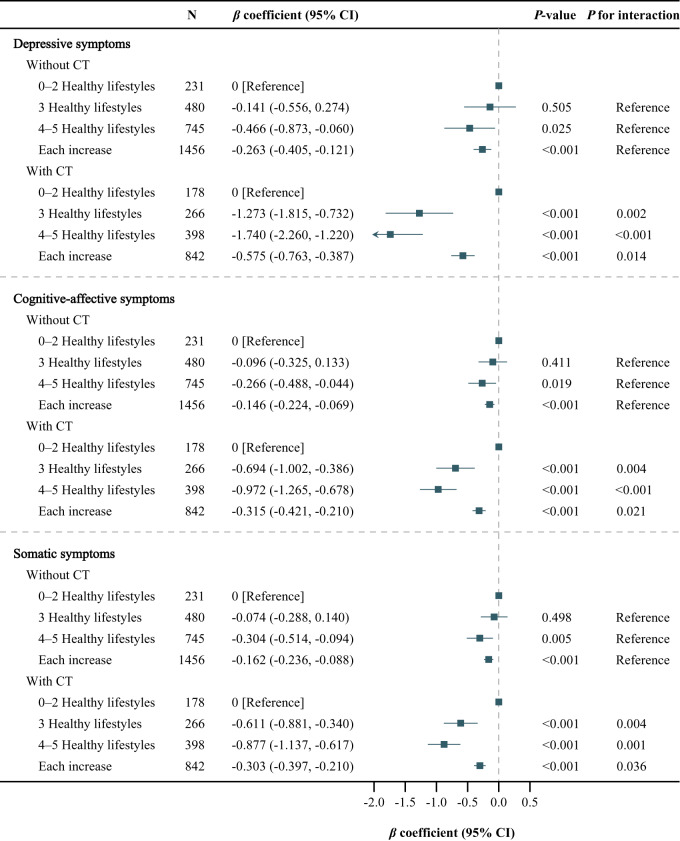
Association of combined lifestyle with depressive symptoms during follow-up, stratified by childhood trauma.

**Figure 3.**
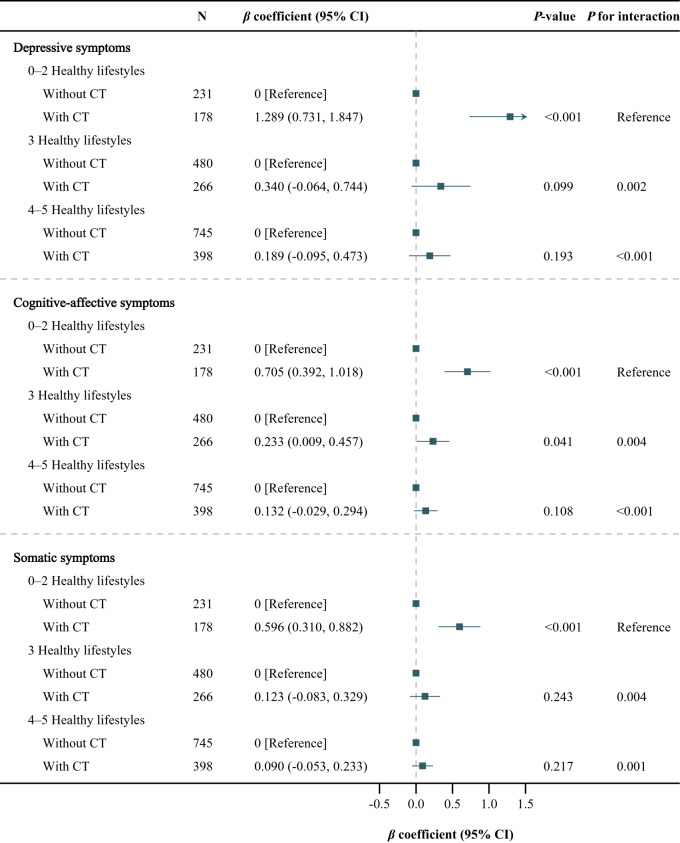
Association of childhood trauma with depressive symptoms during follow-up, stratified by combined lifestyle.

There existed a significant synergistic interaction between an overall healthy lifestyle and the absence of CT (Fig. 2, all *P* for interaction < 0.05). The CT-stratified analysis showed that compared with 0–2 healthy lifestyles, 3 healthy lifestyles were associated with milder depressive (*β* coefficient, −1.273 [95% CI, −1.815, −0.732]), cognitive-affective (*β* coefficient, −0.694 [95% CI, −1.002, −0.386]), and somatic (*β* coefficient, −0.611 [95% CI, −0.881, −0.340]) symptoms in participants with CT, but not in those without CT. Moreover, 4–5 healthy lifestyles were associated with milder depressive (*β* coefficient, −1.740 [95% CI, −2.260, −1.220]; −0.466 [95% CI, −0.873, −0.060]), cognitive-affective (*β* coefficient, −0.972 [95% CI, −1.265, −0.678]; −0.266 [95% CI, −0.488, −0.044]), and somatic (*β* coefficient, −0.877 [95% CI, −1.137, −0.617]; −0.304 [95% CI, −0.514, −0.094]) symptoms in both participants with and without CT, with a stronger association in those with CT. As shown in Fig. 3, the lifestyle-stratified analysis showed that CT was associated with more severe depressive (*β* coefficient, 1.289 [95% CI, 0.731, 1.847]) and somatic (*β* coefficient, 0.596 [95% CI, 0.310, 0.882]) symptoms among participants with 0–2 healthy lifestyles, but not among those with 3 or 4–5 healthy lifestyles. CT was associated with more severe cognitive-affective symptoms among participants with 0–2 (*β* coefficient, 0.705 [95% CI, 0.392, 1.018]) or 3 (*β* coefficient, 0.233 [95% CI, 0.009 and 0.457]) healthy lifestyles, but not among those with 4–5 healthy lifestyles.

The adjusted covariates included follow-up time (follow-up years from baseline) and baseline factors, including age, sex, educational level, employment status, marital status, household income, the number of chronic diseases, depressive symptoms (for depressive symptoms), cognitive-affective symptoms (for cognitive-affective symptoms) and somatic symptoms (for somatic symptoms).Abbreviations: CT, childhood trauma; CI, confidence interval.

The adjusted covariates included follow-up time (follow-up years from baseline) and baseline factors, including age, sex, educational level, employment status, marital status, household income, the number of chronic diseases, depressive symptoms (for depressive symptoms), cognitive-affective symptoms (for cognitive-affective symptoms), and somatic symptoms (for somatic symptoms).Abbreviations: CT, childhood trauma; CI, confidence interval.

### Joint associations of CT and combined lifestyle with depressive symptoms

**Figure 4.**
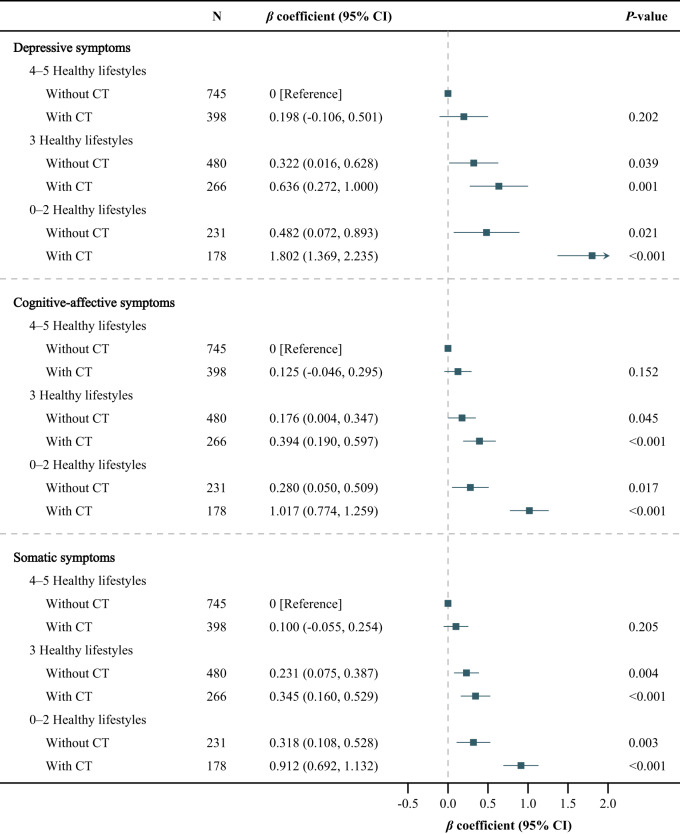
Joint associations of childhood trauma and combined lifestyle with depressive symptoms during follow-up.

Compared with participants with 4–5 healthy lifestyles and without CT (Fig. 4), except for those with 4–5 healthy lifestyles and with CT, others showed more severe depressive, cognitive-affective and somatic symptoms during follow-up. Depressive (*β* coefficient, 1.802 [95% CI, 1.369, 2.235]), cognitive-affective (*β* coefficient, 1.017 [95% CI, 0.774, 1.259]), somatic (*β* coefficient, 0.912 [95% CI, 0.692, 1.132]) symptoms were the most severe among those with 0–2 healthy lifestyles and with CT.

The adjusted covariates included follow-up time (follow-up years from baseline) and baseline factors, including age, sex, educational level, employment status, marital status, household income, the number of chronic diseases, depressive symptoms (for depressive symptoms), cognitive-affective symptoms (for cognitive-affective symptoms), and somatic symptoms (for somatic symptoms).Abbreviations: CT, childhood trauma; CI, confidence interval.

### Sensitivity analyses

The results of the three sensitivity analyses were almost consistent with those of the main analysis (**Table S5–S10** and **Figure S2** in the Supplementary). All statistically significant *β* coefficients still held statistical significance.

## Discussion

In this longitudinal study of 2298 adults with subthreshold depression, better adherence to healthy lifestyles was associated with subsequent milder depressive symptoms and their subtypes, and there existed a significant synergistic interaction between an overall healthy lifestyle and the absence of CT. The CT-stratified analysis showed that healthy lifestyle was associated with subsequent milder depressive symptoms and their subtypes in both adults with and without CT, with a stronger association in those with CT. More importantly, the lifestyle-stratified analysis showed that CT was associated with subsequent more severe depressive symptoms and their subtypes in adults with 0–2 healthy lifestyles, but not in those with 4–5 healthy lifestyles, suggesting that an overall healthy lifestyle might mitigate or even offset the associations of CT with depressive symptoms and their subtypes in adults with subthreshold depression.

### Comparison with other studies

A meta-analysis of five cohort studies has reported that adherence to an overall healthy lifestyle is essential for the primary prevention of depressive symptoms in the general population (Wang *et al.*, 2021). Subsequent cohort studies from multiple countries have also shown similar results in the general population (Cao *et al.*, 2021; Collins *et al.*, 2023; Dabravolskaj *et al.*, 2023; Werneck *et al.*, 2022). Similarly, we found that regardless of the presence of CT, adopting an overall healthy lifestyle was associated with milder depressive symptoms among individuals with subthreshold depression, suggesting that the benefits of an overall healthy lifestyle for depressive symptoms might be generalized to the population with subthreshold depression, a high-risk group for depressive disorders (Zhang *et al.*, 2023a). Lifestyle is a modifiable factor and changing it is low cost. Our findings provide effective, feasible and low-cost strategies for alleviating depressive symptoms in individuals with subthreshold depression, which is of great significance for preventing depressive disorders. Randomized controlled trials are needed to validate our findings in the population with subthreshold depression.

Furthermore, despite the high heterogeneity of depressive symptoms, we found that an overall healthy lifestyle was associated with subsequent milder somatic and cognitive-affective symptoms, reflecting the comprehensive benefits of an overall healthy lifestyle for depressive symptoms. At present, there is a lack of research exploring the associations between combined lifestyle and subtypes of depressive symptoms, with only a few studies evaluating the associations between a single lifestyle (i.e. physical activity and BMI) and subtypes of depressive symptoms in the general population (Chu *et al.*, 2023; Wu *et al.*, 2024). Therefore, further studies are needed to validate our findings across different countries and populations.

CT is widely recognized as a risk factor for depressive symptoms and their subtypes (Humphreys *et al.*, 2020; Iob *et al.*, 2020b, 2021). Our longitudinal study also showed similar findings. Importantly, CT cannot be changed once it occurs and its adverse effects may persist over a lifetime. We found that an overall healthy lifestyle mitigated the associations of CT with subsequent depressive symptoms and their subtypes. Interestingly, stratified analyses of combined lifestyle showed that CT was associated with more severe depressive symptoms and their subtypes among participants with 0–2 healthy lifestyles, but not among those with 4–5 healthy lifestyles, indicating that adherence to an adequate healthy lifestyle might offset the adverse effects of CT on depressive symptoms in adults with subthreshold depression. A relevant biological mechanism might be that CT leads to depressive symptoms primarily by causing the dysregulation of the HPA axis and immune inflammation (Humphreys *et al.*, 2020), whereas adherence to an overall healthy lifestyle is beneficial in maintaining the homeostasis of the HPA axis and immune inflammation, thus an overall healthy lifestyle might mitigate the adverse effects of CT on depressive symptoms (Lopresti *et al.*, 2013). Our findings provide preliminary clues as to how individuals with CT can escape or alleviate the adverse effects of CT on depressive symptoms. To date, few studies have been conducted to target the modifying role of combined lifestyle in the association between CT and depressive symptoms. Several cross-sectional studies of the general population have found that a single lifestyle might modify the association between CT and depressive symptoms, such as physical activity (Boisgontier *et al.*, 2020; Royer and Wharton, 2022), drinking status (Rice *et al.*, 2021), sleep (Masuya *et al.*, 2024) and BMI (Ramirez and Milan, 2016; Zhang *et al.*, 2023b), which to some extent supports our findings. Future studies are needed to verify that an overall healthy lifestyle can alleviate the association between CT and depressive symptoms, and to elucidate the biological mechanisms involved.

### Strengths and limitations

The advantage of this study is that the 3.5-year prospective cohort study design allowed for the identification of temporality between lifestyles and depressive symptoms. In addition, we constructed an overall healthy lifestyle score to comprehensively evaluate the complex associations of lifestyle with depressive symptoms and their subtypes. Nevertheless, several potential limitations should also be noted. First, all variables were collected through the self-reported questionnaire, so reporting bias was inevitable. Second, the retrospective assessment of CT might lead to recall bias. Third, we did not collect information on the specific age at which CT occurred, so we cannot further explore the association between CT at different ages and depressive symptoms. Fourth, since the information on diet was not collected in the SDC study, we did not include diet in healthy lifestyle scores. Thus, our findings cannot suggest whether a healthy lifestyle, including diet, is associated with milder depressive symptoms. Fifth, since this study only involved community residents in Shenzhen, China, the findings need to be carefully extrapolated to other regions in China or other countries. Finally, due to the nature of observational studies, the impact of unmeasured confounding factors (e.g. genotype) on the results cannot be eliminated, hindering the determination of the causal association.

## Conclusions

In this 3.5-year longitudinal study of adults with subthreshold depression, better adherence to healthy lifestyles, including no current drinking, no current smoking, regular physical exercise, optimal sleep duration and no obesity, was associated with subsequent milder depressive symptoms and their subtypes, with a stronger association in adults with CT than those without CT. Furthermore, better adherence to healthy lifestyles significantly mitigated the CT-induced exacerbation of depressive symptoms and their subtypes. Our findings emphasize the benefits of adherence to an overall healthy lifestyle for adults with subthreshold depression, especially those with CT.

## Supporting information

10.1017/S2045796025100127.sm001 Li et al. supplementary material Li et al. supplementary material

## Data Availability

The datasets used in the current study are available from the corresponding author on reasonable request.

## Abbreviations

BMI: body mass index
CI: confidence interval
CT: childhood trauma
CTQ-SF: Childhood Trauma Questionnaire-Short Form
PHQ-9: Patient Health Questionnaire-9
HPA: hypothalamic-pituitary-adrenal
SD: standard deviation
SDC: Subthreshold Depression Cohort

## Supplementary material

The supplementary material for this article can be found at https://doi.org/10.1017/S2045796025100127.
