# Supplementary material for: Combined lifestyle, childhood trauma and depressive symptoms in adults with subthreshold depression: a prospective cohort study
Source: Epidemiol Psychiatr Sci. 2025 Jul 15;34:e39. doi: 10.1017/S2045796025100127 (PMC12281045; doi:10.1017/S2045796025100127)

**Supplementary Materials**

**Title:** Combined Lifestyles, Childhood Trauma, and Depressive Symptoms in Adults with Subthreshold Depression: A Prospective Cohort Study

[**Table S1.** The numbers (percentages) of participants with missing values for covariates. 1](#_Toc195865330)

[**Table S2.** Associations between individual lifestyle and depressive symptoms during follow-up. 2](#_Toc195865331)

[**Table S3.** Baseline characteristics of total participants and those included in analyses. 4](#_Toc195865332)

[**Table S4.** Modifying role of age in the association between CT and depressive symptoms during follow-up 5](#_Toc195865333)

[**Table S5.** Sensitivity analysis 1: association of weighted healthy lifestyle scores with depressive symptoms during follow-up. 6](#_Toc195865334)

[**Table S6.** Sensitivity analysis 2: association between the individual lifestyle and depressive symptoms during follow-up after sequentially excluding each healthy lifestyle. 7](#_Toc195865335)

[**Table S7.** Sensitivity analysis 3: individual association of childhood trauma and combined lifestyle with depressive symptoms during follow-up after excluding participants with missing values for covariates. 10](#_Toc195865336)

[**Table S8.** Sensitivity analysis 3: association of childhood trauma with depressive symptoms during follow-up, stratified by combined lifestyle after excluding participants with missing values for covariates. 11](#_Toc195865337)

[**Table S9.** Sensitivity analysis 3: association of combined lifestyle with depressive symptoms during follow-up, stratified by CT after excluding participants with missing values for covariates. 12](#_Toc195865338)

[**Table S10.** Sensitivity analysis 3: joint associations of CT and combined lifestyle with depressive symptoms during follow-up after excluding participants with missing values for covariates. 13](#_Toc195865339)

[**Figure S1.** Inclusion and exclusion process of participants in this study. 14](#_Toc195865340)

[**Figure S2.** Sensitivity analysis 3: dose-response associations between combined lifestyle and depressive symptoms during follow-up after excluding participants with missing values for covariates. 15](#_Toc195865341)

| Table S1. The numbers (percentages) of participants with missing values for covariates. | | |
| --- | --- | --- |
| **Covariate** | **Number** | **Percentage, %** |
| Educational level | 4 | 0.17 |
| Employment status | 2 | 0.09 |
| Household income | 5 | 0.22 |

| Table S2. Associations between individual lifestyle and depressive symptoms during follow-up. | | | |
| --- | --- | --- | --- |
|  | **N** | ***β* coefficient (95% CI)** | ***P*-value** |
| **Depressive symptoms** |  |  |  |
| Current not smoking |  |  |  |
| No | 358 | 0 [reference] |  |
| Yes | 1940 | -0.372 (-0.592, -0.152) | 0.001 |
| Current not drinking |  |  |  |
| No | 735 | 0 [reference] |  |
| Yes | 1563 | -0.347 (-0.690, -0.004) | 0.047 |
| Regular physical exercise |  |  |  |
| No | 1323 | 0 [reference] |  |
| Yes | 975 | -0.452 (-0.698, -0.206) | <0.001 |
| Optimal sleep duration (7 to <9 hours) |  |  |  |
| No | 1110 | 0 [reference] |  |
| Yes | 1188 | -0.267 (-0.488, -0.046) | 0.018 |
| Body mass index <28 kg/m^2^ |  |  |  |
| No | 142 | 0 [reference] |  |
| Yes | 2156 | -0.672 (-1.123, -0.220) | 0.004 |
| **Cognitive-affective symptoms** |  |  |  |
| Current not smoking |  |  |  |
| No | 358 | 0 [reference] |  |
| Yes | 1940 | -0.214 (-0.337, -0.091) | 0.001 |
| Current not drinking |  |  |  |
| No | 735 | 0 [reference] |  |
| Yes | 1563 | -0.183 (-0.375, 0.010) | 0.063 |
| Regular physical exercise |  |  |  |
| No | 1323 | 0 [reference] |  |
| Yes | 975 | -0.223 (-0.361, -0.085) | 0.002 |
| Optimal sleep duration (7 to <9 hours) |  |  |  |
| No | 1110 | 0 [reference] |  |
| Yes | 1188 | -0.150 (-0.273, -0.028) | 0.016 |
| Body mass index <28 kg/m^2^ |  |  |  |
| No | 142 | 0 [reference] |  |
| Yes | 2156 | -0.470 (-0.724, -0.217) | <0.001 |
| **Somatic symptoms** |  |  |  |
| Current not smoking |  |  |  |
| No | 358 | 0 [reference] |  |
| Yes | 1940 | -0.198 (-0.310, -0.087) | <0.001 |
| Current not drinking |  |  |  |
| No | 735 | 0 [reference] |  |
| Yes | 1563 | -0.198 (-0.373, -0.024) | 0.026 |
| Regular physical exercise |  |  |  |
| No | 1323 | 0 [reference] |  |
| Yes | 975 | -0.235 (-0.360, -0.110) | <0.001 |
| Optimal sleep duration (7 to <9 hours) |  |  |  |
| No | 1110 | 0 [reference] |  |
| Yes | 1188 | -0.214 (-0.327, -0.100) | <0.001 |
| Body mass index <28 kg/m^2^ |  |  |  |
| No | 142 | 0 [reference] |  |
| Yes | 2156 | -0.260 (-0.490, -0.030) | 0.027 |
| Lifestyles were mutually adjusted in the models. Other adjusted covariates included follow-up time (follow-up years from baseline) and baseline factors, including age, sex, educational level, employment status, marital status, household income, the number of chronic diseases, childhood trauma, depressive symptoms (for depressive symptoms), cognitive-affective symptoms (for cognitive-affective symptoms), and somatic symptoms (for somatic symptoms).  Abbreviations: CI, confidence interval. | | | |

| Table S3. Baseline characteristics of total participants and those included in analyses. | | | | |
| --- | --- | --- | --- | --- |
| **Characteristic^a^** | **Total (n=2306)** | **Included (n=****2298)** | ***P*-value^b^** | |
| Age, mean (SD), years | 40.3 (11.1) | 40.3 (11.1) | 0.967 | |
| Male | 870 (37.7) | 866 (37.7) | 0.976 | |
| Educational level |  |  | 0.998 | |
| Junior high school or below | 287 (12.5) | 286 (12.5) |  | |
| Senior high school | 536 (23.3) | 536 (23.4) |  | |
| College or above | 1479 (64.3) | 1472 (64.2) |  | |
| Employment status |  |  | 1.000 | |
| Employed | 1821 (79.0) | 1816 (79.1) |  | |
| Unemployed | 77 (3.3) | 77 (3.4) |  | |
| Retired | 158 (6.9) | 156 (6.8) |  | |
| Others | 248 (10.8) | 247 (10.8) |  | |
| Marital status |  |  | 0.998 | |
| Married | 626 (27.1) | 623 (27.1) |  | |
| Unmarried | 1590 (69.0) | 1586 (69.0) |  | |
| Divorced/widowed | 90 (3.9) | 89 (3.9) |  | |
| Household income |  |  | 0.997 | |
| <10 000 yuan/month | 1059 (46.0) | 1055 (46.0) |  | |
| 10 000–19 999 yuan/month | 689 (29.9) | 685 (29.9) |  | |
| ≥20 000 yuan/month | 553 (24.0) | 553 (24.1) |  | |
| Lifestyles |  |  |  | |
| Current not smoking | 1947 (84.5) | 1940 (84.4) | 0.965 | |
| Current not drinking | 1566 (68.0) | 1563 (68.0) | 0.993 | |
| Regular physical exercise | 980 (42.5) | 975 (42.4) | 0.942 | |
| Optimal sleep duration (7 to <9 hours) | 1192 (51.7) | 1188 (51.7) | 0.997 | |
| Body mass index <28 kg/m^2^ | 2161 (93.8) | 2156 (93.8) | 0.924 | |
| Number of chronic diseases |  |  | 0.999 | |
| 0 | 1931 (83.7) | 1923 (83.7) |  | |
| 1 | 332 (14.4) | 332 (14.4) |  | |
| ≥2 | 43 (1.9) | 43 (1.9) |  | |
| With childhood trauma | 847 (36.7) | 842 (36.6) | 0.950 | |
| Depressive symptom score, mean (SD) | 8.7 (4.3) | 8.7 (4.3) | 0.915 | |
| Cognitive-affective symptom score, mean (SD) | 4.2 (2.7) | 4.2 (2.7) | 0.923 | |
| Somatic symptom score, mean (SD) | 4.5 (2.2) | 4.5 (2.2) | 0.927 | |
| ^a^ Unless otherwise indicated, data are expressed as No. (%) of participants. Percentages have been rounded, so the total may not be 100%.  ^b^ Two independent sample *t*-tests were used to compare the means of continuous variables. Pearson Chi-squared tests were performed to compare the distribution of categorical variables.  Abbreviation: SD, standard deviation. | | | |  |

| Table S4. Modifying role of age in the association between CT and depressive symptoms during follow-up | | | |
| --- | --- | --- | --- |
| **Outcome** | **Interaction term** | ***β* coefficient (95% CI) for interaction term** | ***P*-value for interaction term** |
| Depressive symptoms | CT*age | -0.014 (-0.033, 0.006) | 0.162 |
| Cognitive-affective symptoms | CT*age | -0.011 (-0.022, 0.001) | 0.055 |
| Somatic symptoms | CT*age | -0.004 (-0.014, 0.006) | 0.451 |
| Models included CT, age, CT×age, follow-up time (follow-up years from baseline), and baseline factors, including sex, educational level, employment status, marital status, household income, the number of chronic diseases, combined lifestyle, depressive symptoms (for depressive symptoms), cognitive-affective symptoms (for cognitive-affective symptoms), and somatic symptoms (for somatic symptoms).  Abbreviations: CT, childhood trauma; CI, confidence interval. | | | |

| Table S5. Sensitivity analysis 1: association of weighted healthy lifestyle scores with depressive symptoms during follow-up. | | | |
| --- | --- | --- | --- |
|  | **N** | ***β* coefficient (95% CI)** | ***P*-value** |
| **Depressive symptoms** |  |  |  |
| Tertile 1 of scores (unfavorable) | 767 | 0 [reference] |  |
| Tertile 2 of scores (intermediate) | 512 | -0.576 (-0.875, -0.277) | <0.001 |
| Tertile 3 of scores (favorable) | 1019 | -0.776 (-1.036, -0.516) | <0.001 |
| Each increase in scores | 2298 | -1.000 (-1.284, -0.716) | <0.001 |
| **Cognitive-affective symptoms** |  |  |  |
| Tertile 1 of scores (unfavorable) | 767 | 0 [reference] |  |
| Tertile 2 of scores (intermediate) | 512 | -0.285 (-0.453, -0.117) | 0.001 |
| Tertile 3 of scores (favorable) | 1019 | -0.422 (-0.568, -0.277) | <0.001 |
| Each increase in scores | 2298 | -1.000 (-1.279, -0.721) | <0.001 |
| **Somatic symptoms** |  |  |  |
| Tertile 1 of scores (unfavorable) | 730 | 0 [reference] |  |
| Tertile 2 of scores (intermediate) | 818 | -0.305 (-0.442, -0.168) | <0.001 |
| Tertile 3 of scores (favorable) | 750 | -0.456 (-0.599, -0.313) | <0.001 |
| Each increase in scores | 2298 | -1.000 (-1.263, -0.737) | <0.001 |
| The adjusted covariates included follow-up time (follow-up years from baseline) and baseline factors, including age, sex, educational level, employment status, marital status, household income, the number of chronic diseases, childhood trauma, depressive symptoms (for depressive symptoms), cognitive-affective symptoms (for cognitive-affective symptoms), and somatic symptoms (for somatic symptoms). | | | |

| Table S6. Sensitivity analysis 2: association between the individual lifestyle and depressive symptoms during follow-up after sequentially excluding each healthy lifestyle. | | | |
| --- | --- | --- | --- |
|  | **N** | ***β* coefficient (95% CI)** | ***P*-value** |
| **Depressive symptoms** |  |  |  |
| Not including physical exercise |  |  |  |
| 0–2 Healthy lifestyles | 600 | 0 [reference] |  |
| 3 Healthy lifestyles | 975 | -0.454 (-0.731, -0.176) | 0.001 |
| 4 Healthy lifestyles | 723 | -0.766 (-1.070, -0.461) | <0.001 |
| Each increase | 2298 | -0.386 (-0.514, -0.257) | <0.001 |
| Not including smoking status |  |  |  |
| 0–2 Healthy lifestyles | 1070 | 0 [reference] |  |
| 3 Healthy lifestyles | 906 | -0.511 (-0.749, -0.274) | <0.001 |
| 4 Healthy lifestyles | 322 | -0.727 (-1.060, -0.395) | <0.001 |
| Each increase | 2298 | -0.382 (-0.510, -0.255) | <0.001 |
| Not including smoking status |  |  |  |
| 0–2 Healthy lifestyles | 875 | 0 [reference] |  |
| 3 Healthy lifestyles | 1016 | -0.408 (-0.652, -0.165) | 0.001 |
| 4 Healthy lifestyles | 407 | -0.692 (-1.008, -0.375) | <0.001 |
| Each increase | 2298 | -0.357 (-0.491, -0.223) | <0.001 |
| Not including sleep duration |  |  |  |
| 0–2 Healthy lifestyles | 631 | 0 [reference] |  |
| 3 Healthy lifestyles | 1103 | -0.654 (-0.922, -0.387) | <0.001 |
| 4 Healthy lifestyles | 564 | -0.935 (-1.248, -0.622) | <0.001 |
| Each increase | 2298 | -0.421 (-0.551, -0.290) | <0.001 |
| Not including body mass index |  |  |  |
| 0–2 Healthy lifestyles | 1107 | 0 [reference] |  |
| 3 Healthy lifestyles | 884 | -0.436 (-0.673, -0.200) | <0.001 |
| 4 Healthy lifestyles | 307 | -0.718 (-1.053, -0.382) | <0.001 |
| Each increase | 2298 | -0.360 (-0.477, -0.244) | <0.001 |
| **Cognitive-affective symptoms** |  |  |  |
| Not including physical exercise |  |  |  |
| 0–2 Healthy lifestyles | 600 | 0 [reference] |  |
| 3 Healthy lifestyles | 975 | -0.229 (-0.385, -0.073) | 0.004 |
| 4 Healthy lifestyles | 723 | -0.408 (-0.578, -0.239) | <0.001 |
| Each increase | 2298 | -0.211 (-0.283, -0.140) | <0.001 |
| Not including smoking status |  |  |  |
| 0–2 Healthy lifestyles | 1070 | 0 [reference] |  |
| 3 Healthy lifestyles | 906 | -0.306 (-0.438, -0.174) | <0.001 |
| 4 Healthy lifestyles | 322 | -0.398 (-0.584, -0.213) | <0.001 |
| Each increase | 2298 | -0.216 (-0.287, -0.145) | <0.001 |
| Not including smoking status |  |  |  |
| 0–2 Healthy lifestyles | 875 | 0 [reference] |  |
| 3 Healthy lifestyles | 1016 | -0.265 (-0.401, -0.129) | <0.001 |
| 4 Healthy lifestyles | 407 | -0.388 (-0.563, -0.212) | <0.001 |
| Each increase | 2298 | -0.208 (-0.283, -0.134) | <0.001 |
| Not including sleep duration |  |  |  |
| 0–2 Healthy lifestyles | 631 | 0 [reference] |  |
| 3 Healthy lifestyles | 1103 | -0.368 (-0.518, -0.217) | <0.001 |
| 4 Healthy lifestyles | 564 | -0.511 (-0.687, -0.336) | <0.001 |
| Each increase | 2298 | -0.233 (-0.306, -0.160) | <0.001 |
| Not including body mass index |  |  |  |
| 0–2 Healthy lifestyles | 1107 | 0 [reference] |  |
| 3 Healthy lifestyles | 884 | -0.245 (-0.377, -0.114) | <0.001 |
| 4 Healthy lifestyles | 307 | -0.384 (-0.571, -0.197) | <0.001 |
| Each increase | 2298 | -0.193 (-0.257, -0.128) | <0.001 |
| **Somatic symptoms** |  |  |  |
| Not including physical exercise |  |  |  |
| 0–2 Healthy lifestyles | 600 | 0 [reference] |  |
| 3 Healthy lifestyles | 975 | -0.250 (-0.392, -0.109) | 0.001 |
| 4 Healthy lifestyles | 723 | -0.469 (-0.625, -0.314) | <0.001 |
| Each increase | 2298 | -0.222 (-0.288, -0.157) | <0.001 |
| Not including smoking status |  |  |  |
| 0–2 Healthy lifestyles | 1070 | 0 [reference] |  |
| 3 Healthy lifestyles | 906 | -0.287 (-0.408, -0.167) | <0.001 |
| 4 Healthy lifestyles | 322 | -0.445 (-0.614, -0.276) | <0.001 |
| Each increase | 2298 | -0.218 (-0.283, -0.153) | <0.001 |
| Not including smoking status |  |  |  |
| 0–2 Healthy lifestyles | 875 | 0 [reference] |  |
| 3 Healthy lifestyles | 1016 | -0.228 (-0.353, -0.104) | <0.001 |
| 4 Healthy lifestyles | 407 | -0.438 (-0.600, -0.277) | <0.001 |
| Each increase | 2298 | -0.210 (-0.278, -0.141) | <0.001 |
| Not including sleep duration |  |  |  |
| 0–2 Healthy lifestyles | 631 | 0 [reference] |  |
| 3 Healthy lifestyles | 1103 | -0.324 (-0.460, -0.187) | <0.001 |
| 4 Healthy lifestyles | 564 | -0.494 (-0.653, -0.335) | <0.001 |
| Each increase | 2298 | -0.216 (-0.283, -0.150) | <0.001 |
| Not including body mass index |  |  |  |
| 0–2 Healthy lifestyles | 1107 | 0 [reference] |  |
| 3 Healthy lifestyles | 884 | -0.266 (-0.386, -0.145) | <0.001 |
| 4 Healthy lifestyles | 307 | -0.450 (-0.621, -0.280) | <0.001 |
| Each increase | 2298 | -0.213 (-0.272, -0.154) | <0.001 |
| Lifestyles were mutually adjusted in the models, Other adjusted covariates included follow-up time (follow-up years from baseline) and baseline factors, including age, sex, educational level, employment status, marital status, household income, the number of chronic diseases, childhood trauma, depressive symptoms (for depressive symptoms), cognitive-affective symptoms (for cognitive-affective symptoms), and somatic symptoms (for somatic symptoms). | | | |

| Table S7. Sensitivity analysis 3: individual association of childhood trauma and combined lifestyle with depressive symptoms during follow-up after excluding participants with missing values for covariates. | | | |
| --- | --- | --- | --- |
|  | **N** | ***β* coefficient (95% CI)** | ***P*-value** |
| **Depressive symptoms** |  |  |  |
| Combined lifestyle |  |  |  |
| 0–2 Healthy lifestyles | 408 | 0 [reference] |  |
| 3 Healthy lifestyles | 744 | -0.622 (-0.947, -0.297) | <0.001 |
| 4–5 Healthy lifestyles | 1140 | -0.991 (-1.308, -0.674) | <0.001 |
| Each increase | 2292 | -0.382 (-0.494, -0.270) | <0.001 |
| Childhood trauma |  |  |  |
| No | 1452 | 0 [reference] |  |
| Yes | 840 | 0.445 (0.228, 0.661) | <0.001 |
| **Cognitive-affective symptoms** |  |  |  |
| Combined lifestyle |  |  |  |
| 0–2 Healthy lifestyles | 408 | 0 [reference] |  |
| 3 Healthy lifestyles | 744 | -0.344 (-0.526, -0.162) | <0.001 |
| 4–5 Healthy lifestyles | 1140 | -0.557 (-0.733, -0.380) | <0.001 |
| Each increase | 2292 | -0.212 (-0.274, -0.150) | <0.001 |
| Childhood trauma |  |  |  |
| No | 1452 | 0 [reference] |  |
| Yes | 840 | 0.268 (0.146, 0.390) | <0.001 |
| **Somatic symptoms** |  |  |  |
| Combined lifestyle |  |  |  |
| 0–2 Healthy lifestyles | 408 | 0 [reference] |  |
| 3 Healthy lifestyles | 744 | -0.306 (-0.472, -0.141) | <0.001 |
| 4–5 Healthy lifestyles | 1140 | -0.545 (-0.707, -0.384) | <0.001 |
| Each increase | 2292 | -0.216 (-0.274, -0.159) | <0.001 |
| Childhood trauma |  |  |  |
| No | 1452 | 0 [reference] |  |
| Yes | 840 | 0.197 (0.087, 0.307) | <0.001 |
| Combined lifestyle and childhood trauma were mutually adjusted in the models. Other adjusted covariates included follow-up time (follow-up years from baseline) and baseline factors, including age, sex, educational level, employment status, marital status, household income, the number of chronic diseases, depressive symptoms (for depressive symptoms), cognitive-affective symptoms (for cognitive-affective symptoms), and somatic symptoms (for somatic symptoms). | | | |

| Table S8. Sensitivity analysis 3: association of childhood trauma with depressive symptoms during follow-up, stratified by combined lifestyle after excluding participants with missing values for covariates. | | | | |
| --- | --- | --- | --- | --- |
|  | **N** | ***β* coefficient (95% CI)** | ***P*-value** | ***P* for interaction** |
| **Depressive symptoms** |  |  |  |  |
| 0–2 Healthy lifestyles |  |  |  |  |
| Without CT | 230 | 0 [reference] |  |  |
| With CT | 178 | 1.286 (0.727, 1.845) | <0.001 | Reference |
| 3 Healthy lifestyles |  |  |  |  |
| Without CT | 479 | 0 [reference] |  |  |
| With CT | 265 | 0.348 (-0.057, 0.753) | 0.092 | 0.002 |
| 4–5 Healthy lifestyles |  |  |  |  |
| Without CT | 743 | 0 [reference] |  |  |
| With CT | 397 | 0.195 (-0.090, 0.480) | 0.179 | <0.001 |
| **Cognitive-affective symptoms** |  |  |  |  |
| 0–2 Healthy lifestyles |  |  |  |  |
| Without CT | 230 | 0 [reference] |  |  |
| With CT | 178 | 0.703 (0.389, 1.016) | <0.001 | Reference |
| 3 Healthy lifestyles |  |  |  |  |
| Without CT | 479 | 0 [reference] |  |  |
| With CT | 265 | 0.238 (0.013, 0.463) | 0.038 | 0.005 |
| 4–5 Healthy lifestyles |  |  |  |  |
| Without CT | 743 | 0 [reference] |  |  |
| With CT | 397 | 0.136 (-0.026, 0.298) | 0.100 | <0.001 |
| **Somatic symptoms** |  |  |  |  |
| 0–2 Healthy lifestyles |  |  |  |  |
| Without CT | 230 | 0 [reference] |  |  |
| With CT | 178 | 0.596 (0.310, 0.883) | <0.001 | Reference |
| 3 Healthy lifestyles |  |  |  |  |
| Without CT | 479 | 0 [reference] |  |  |
| With CT | 265 | 0.127 (-0.080, 0.334) | 0.230 | 0.004 |
| 4–5 Healthy lifestyles |  |  |  |  |
| Without CT | 743 | 0 [reference] |  |  |
| With CT | 397 | 0.092 (-0.051, 0.236) | 0.207 | 0.001 |
| The adjusted covariates included follow-up time (follow-up years from baseline) and baseline factors, including age, sex, educational level, employment status, marital status, household income, the number of chronic diseases, depressive symptoms (for depressive symptoms), cognitive-affective symptoms (for cognitive-affective symptoms), and somatic symptoms (for somatic symptoms).  Abbreviations: CT, childhood trauma; CI, confidence interval. | | | | |

| Table S9. Sensitivity analysis 3: association of combined lifestyle with depressive symptoms during follow-up, stratified by CT after excluding participants with missing values for covariates. | | | | |
| --- | --- | --- | --- | --- |
|  | **N** | ***β* coefficient (95% CI)** | ***P*-value** | ***P* for interaction** |
| **Depressive symptoms** |  |  |  |  |
| Without CT |  |  |  |  |
| 0–2 Healthy lifestyles | 230 | 0 [reference] |  |  |
| 3 Healthy lifestyles | 479 | -0.146 (-0.562, 0.270) | 0.492 | Reference |
| 4–5 Healthy lifestyles | 743 | -0.475 (-0.883, -0.066) | 0.023 | Reference |
| Each increase | 1452 | -0.265 (-0.408, -0.123) | <0.001 | Reference |
| With CT |  |  |  |  |
| 0–2 Healthy lifestyles | 178 | 0 [reference] |  |  |
| 3 Healthy lifestyles | 265 | -1.272 (-1.815, -0.730) | <0.001 | 0.002 |
| 4–5 Healthy lifestyles | 397 | -1.737 (-2.258, -1.216) | <0.001 | <0.001 |
| Each increase | 840 | -0.574 (-0.762, -0.386) | <0.001 | 0.015 |
| **Cognitive-affective symptoms** |  |  |  |  |
| Without CT | 230 |  |  |  |
| 0–2 Healthy lifestyles | 479 | 0 [reference] |  |  |
| 3 Healthy lifestyles | 743 | -0.099 (-0.328, 0.130) | 0.398 | Reference |
| 4–5 Healthy lifestyles | 1452 | -0.271 (-0.494, -0.048) | 0.017 | Reference |
| Each increase |  | -0.148 (-0.226, -0.070) | <0.001 | Reference |
| With CT | 178 |  |  |  |
| 0–2 Healthy lifestyles | 265 | 0 [reference] |  |  |
| 3 Healthy lifestyles | 397 | -0.693 (-1.002, -0.385) | <0.001 | 0.005 |
| 4–5 Healthy lifestyles | 840 | -0.971 (-1.265, -0.677) | <0.001 | <0.001 |
| Each increase | 842 | -0.315 (-0.421, -0.209) | <0.001 | 0.022 |
| **Somatic symptoms** |  |  |  |  |
| Without CT | 230 |  |  |  |
| 0–2 Healthy lifestyles | 479 | 0 [reference] |  |  |
| 3 Healthy lifestyles | 743 | -0.076 (-0.291, 0.139) | 0.488 | Reference |
| 4–5 Healthy lifestyles | 1452 | -0.307 (-0.518, -0.095) | 0.004 | Reference |
| Each increase |  | -0.163 (-0.237, -0.089) | <0.001 | Reference |
| With CT | 178 |  |  |  |
| 0–2 Healthy lifestyles | 265 | 0 [reference] |  |  |
| 3 Healthy lifestyles | 397 | -0.610 (-0.881, -0.339) | <0.001 | 0.004 |
| 4–5 Healthy lifestyles | 840 | -0.877 (-1.137, -0.616) | <0.001 | 0.001 |
| Each increase | 842 | -0.303 (-0.397, -0.209) | <0.001 | 0.037 |
| The adjusted covariates included follow-up time (follow-up years from baseline) and baseline factors, including age, sex, educational level, employment status, marital status, household income, the number of chronic diseases, depressive symptoms (for depressive symptoms), cognitive-affective symptoms (for cognitive-affective symptoms), and somatic symptoms (for somatic symptoms).  Abbreviations: CT, childhood trauma; CI, confidence interval. | | | | |

| Table S10. Sensitivity analysis 3: joint associations of CT and combined lifestyle with depressive symptoms during follow-up after excluding participants with missing values for covariates. | | | |
| --- | --- | --- | --- |
|  | **N** | ***β* coefficient (95% CI)** | ***P*-value** |
| **Depressive symptoms** |  |  |  |
| 4–5 Healthy lifestyles |  |  |  |
| Without CT | 743 | 0 [reference] |  |
| With CT | 397 | 0.205 (-0.099, 0.510) | 0.186 |
| 3 Healthy lifestyles |  |  |  |
| Without CT | 479 | 0.324 (0.017, 0.630) | 0.039 |
| With CT | 265 | 0.645 (0.279, 1.010) | 0.001 |
| 0–2 Healthy lifestyles |  |  |  |
| Without CT | 230 | 0.489 (0.077, 0.901) | 0.020 |
| With CT | 178 | 1.809 (1.375, 2.243) | <0.001 |
| **Cognitive-affective symptoms** |  |  |  |
| 4–5 Healthy lifestyles |  |  |  |
| Without CT | 743 | 0 [reference] |  |
| With CT | 397 | 0.129 (-0.042, 0.300) | 0.140 |
| 3 Healthy lifestyles |  |  |  |
| Without CT | 479 | 0.177 (0.005, 0.349) | 0.043 |
| With CT | 265 | 0.398 (0.194, 0.603) | <0.001 |
| 0–2 Healthy lifestyles |  |  |  |
| Without CT | 230 | 0.284 (0.054, 0.514) | 0.016 |
| With CT | 178 | 1.020 (0.778, 1.263) | <0.001 |
| **Somatic symptoms** |  |  |  |
| 4–5 Healthy lifestyles |  |  |  |
| Without CT | 743 | 0 [reference] |  |
| With CT | 397 | 0.103 (-0.052, 0.258) | 0.192 |
| 3 Healthy lifestyles |  |  |  |
| Without CT | 479 | 0.232 (0.075, 0.388) | 0.004 |
| With CT | 265 | 0.349 (0.164, 0.535) | <0.001 |
| 0–2 Healthy lifestyles |  |  |  |
| Without CT | 230 | 0.321 (0.110, 0.532) | 0.003 |
| With CT | 178 | 0.916 (0.696, 1.135) | <0.001 |
| The adjusted covariates included follow-up time (follow-up years from baseline) and baseline factors, including age, sex, educational level, employment status, marital status, household income, the number of chronic diseases, depressive symptoms (for depressive symptoms), cognitive-affective symptoms (for cognitive-affective symptoms), and somatic symptoms (for somatic symptoms).  Abbreviations: CT, childhood trauma; CI, confidence interval. | | | |

Figure S1. Inclusion and exclusion process of participants in this study.


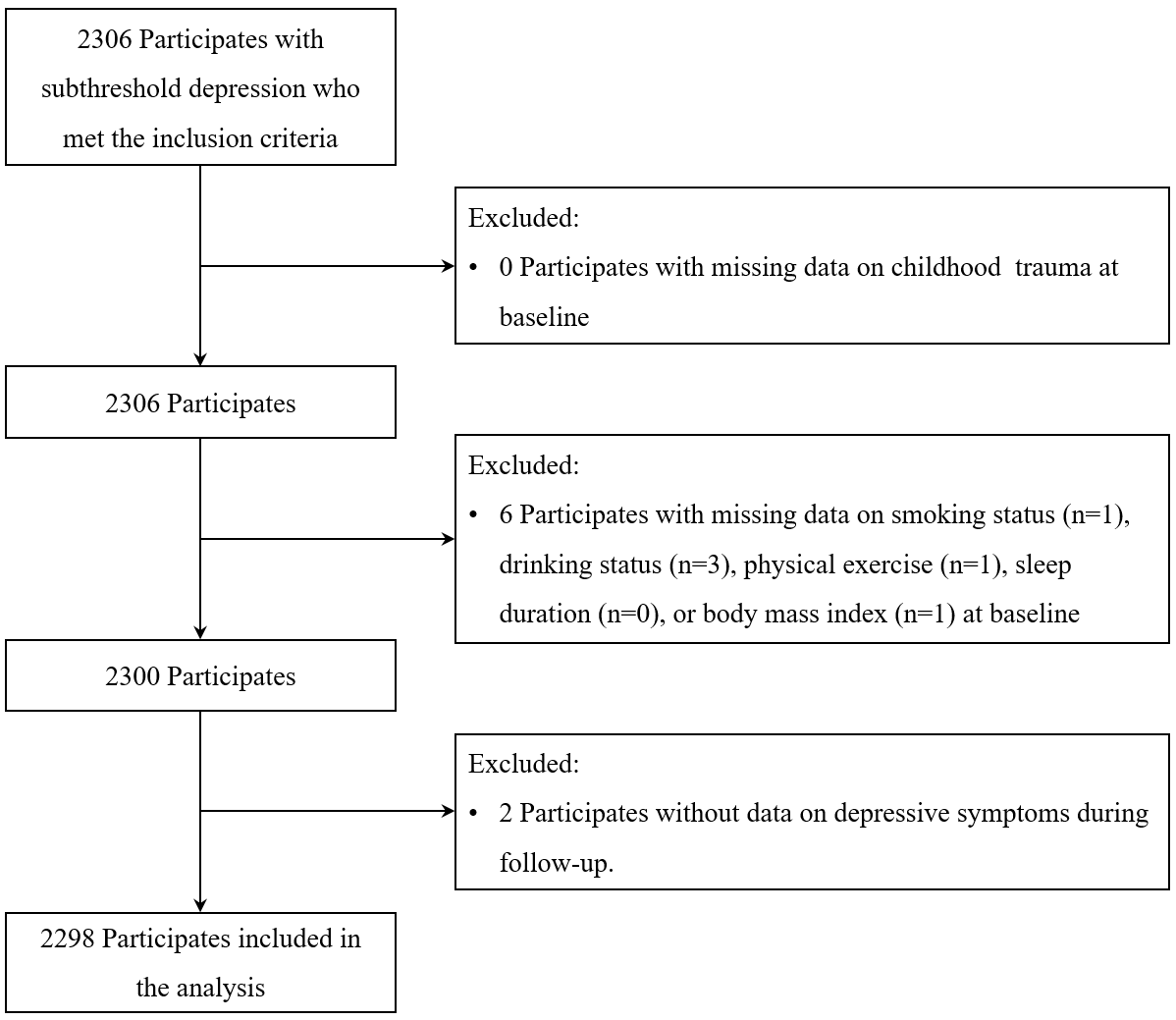


Figure S2. Sensitivity analysis 3: dose-response associations between combined lifestyle and depressive symptoms during follow-up after excluding participants with missing values for covariates.

The solid line and dashed line represent the estimated values and their 95% CI. The adjusted covariates included follow-up time (follow-up years from baseline) and baseline factors, including age, sex, educational level, employment status, marital status, household income, the number of chronic diseases, childhood trauma, depressive symptoms (for depressive symptoms), cognitive-affective symptoms (for cognitive-affective symptoms), and somatic symptoms (for somatic symptoms).

Abbreviations: CI, confidence interval.
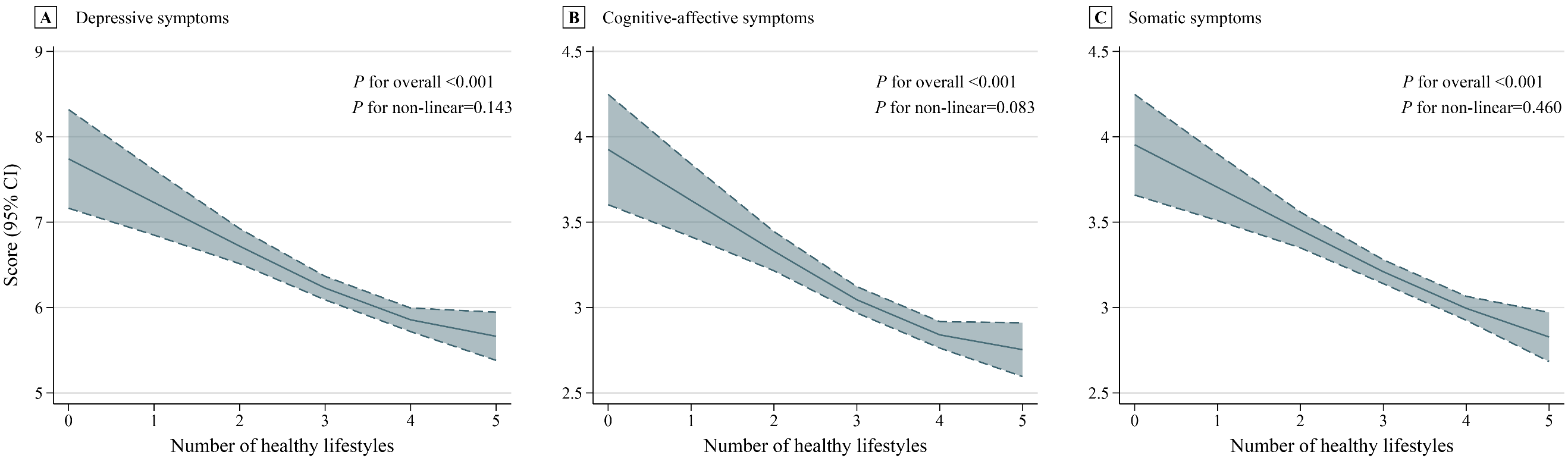

Supplement: Li et al. supplementary material [file S2045796025100127sup001.docx]
